# Developing a Novel Artificial Intelligence Framework to Measure the Balance of Clinical Versus Nonclinical Influences on Posthepatectomy Length of Stay

**DOI:** 10.1245/s10434-025-16942-5

**Published:** 2025-02-05

**Authors:** Kristin Putman, Mohamad El Moheb, Chengli Shen, Russell G. Witt, Samantha M. Ruff, Allan Tsung

**Affiliations:** 1 Department of Surgery, University of Virginia, Charlottesville, VA USA; 2 School of Data Science, University of Virginia, Charlottesville, VA USA

**Keywords:** Hepatectomy, Artificial intelligence, Length of stay, Hepatectomy, Machine learning, Quality improvement

## Abstract

**Background:**

Length of stay (LOS) is a key indicator of posthepatectomy care quality. While clinical factors influencing LOS are identified, the balance between clinical and nonclinical influences remains unquantified. We developed an artificial intelligence (AI) framework to quantify clinical influences on LOS and infer the impact of hard-to-measure nonclinical factors.

**Methods:**

Patients from the 2017 to 2021 ACS NSQIP Hepatectomy-Targeted database were stratified into major and minor hepatectomy groups. A three-tiered model—multivariable linear regression (MLR), random forest (RF), and extreme gradient boosting (XGBoost)—was developed to evaluate the effect of 52 clinical variables on LOS. Models were fine-tuned to maximize clinical variables’ explanatory power, with residual unexplained variability attributed to nonclinical factors. Model performance was measured using *R*^2^ and mean absolute error (MAE).

**Results:**

A total of 21,039 patients (mean age: 60 years; 51% male) were included: 70% underwent minor resection (mean LOS: 5.0 days), and 30% underwent major resection (mean LOS: 6.9 days). Random forest had the best performance, explaining 75% of LOS variability for both groups (*R*^2^: 0.75). The significant improvement in *R*^2^ from MLR to RF suggests significant nonlinear interactions of clinical factors’ impact on LOS. Mean absolute errors were 1.15 and 1.38 days for minor and major resections, indicating that clinical factors could not explain 1.15 to 1.38 days of LOS.

**Conclusions:**

This study is the first to measure the true influence of clinical factors on posthepatectomy LOS, showing that they explain 75% of the variability. Furthermore, it indirectly evaluated the overall impact of hard-to-measure nonclinical factors, revealing that they account for 25% of LOS.

**Supplementary Information:**

The online version contains supplementary material available at 10.1245/s10434-025-16942-5.

Hepatectomy is a complex and resource-intensive procedure that often results in postoperative morbidity and prolonged length of stay (LOS).^1,2^ Development and implementation of enhanced recovery protocols, as well as increased utilization of minimally invasive techniques, have successfully reduced LOS and improved postoperative outcomes.^3–9^ Despite these advancements, LOS after hepatectomy remains variable and unpredictable, suggesting that nonclinical factors may also play a significant role.^10,11^ For example, research has shown that high-volume hospitals performing liver resections achieve shorter LOS by an average of 1.24 days compared with low-volume hospitals, attributed to better resource availability.^12^ Furthermore, several operational and logistical factors, such as surgical start times, implementing effective discharge planning, improving care coordination, and ensuring adequate social support for patients, are crucial factors for reducing LOS.^13–16^

While the influence of nonclinical factors on LOS is acknowledged, their precise impact remains largely unmeasured. This hampers surgeons’ ability to accurately predict and optimize patient LOS, potentially leading to inefficient resource allocation and suboptimal patient care. Evidence from other disciplines indicates that most of the variation in LOS is not clinical in nature, suggesting that future efforts designed to reduce LOS will yield greater returns if nonclinical factors are targeted.^17–20^ Furthermore, insight into the proportion of LOS due to clinical versus nonclinical factors is crucial to interpret benchmark results, because risk-adjustment typically only considers clinical factors. Understanding the reliability of these adjustments and the proportion of LOS explained by unmeasured factors, which are predominantly nonclinical, is essential for accurate performance evaluation.

Two main challenges have historically impeded the ability to quantify the proportion of LOS attributable to clinical versus nonclinical factors. First, while clinical factors are easily identified and effectively captured by most registries, nonclinical factors are more numerous, and difficult to record comprehensively, such as clinical judgement and patient preferences. Logically, because factors can only be clinical or nonclinical, one might assume that accurately measuring the impact of clinical factors would allow for easy deduction of nonclinical factors’ impact. However, this leads to the second challenge: the assumption that available clinical variables can fully explain their contribution to LOS variability. Researchers have traditionally relied on linear models, which often overlook the intricate interactions between patient demographics, comorbidities, and operative details. Consequently, these models fail to fully reflect the complexity of the clinical environment and the true influence of variables of interest, limiting our ability to extract the full extent to which clinical variables contribute to LOS.

Artificial intelligence (AI) models offer a potential solution to these challenges by uncovering complex relationships between variables. This approach could potentially quantify clinical factors’ total contribution to LOS and, for the first time, enable inference of the overall impact of traditionally hard-to-measure nonclinical factors. Such an achievement would provide unprecedented insight into the true balance of clinical and nonclinical influences on LOS, potentially revolutionizing our approach to LOS management and benchmarks in healthcare. In this study, we created a novel AI framework to accurately quantify the proportion of LOS variability attributable to clinical factors in major and minor liver resections. By utilizing residual confounding, we sought to infer the influence of nonclinical and system-related factors.

## Methods

This study was exempted from informed consent by the University of Virginia Institutional Review Board.

### Data Source

In this retrospective cohort study, adult patients (age ≥ 18 years) who underwent hepatectomy were selected from the American College of Surgeons National Surgical Quality Improvement Program (ACS-NSQIP) 2017–2021 database and stratified into minor (Current Procedural Terminology codes [CPT]: 47120) versus major resection cohorts (CPT: 47122, 47125, 47130). Hepatectomy-specific variables were retrieved from ACS-NSQIP Hepatectomy Targeted PUF by matching on surgery case identification number. Patients were excluded if they were transferred from an outside hospital, expired during hospitalization, underwent multiple procedures, or had outlying LOS (greater than 99th percentile).

### Model Development

Three models were built for each surgery group: (1) multivariable linear regression (MLR), (2) random forest (RF), and (3) extreme gradient boosting (XGBoost). After examining both the main and targeted ACS-NSQIP data, a total of 52 clinical and patient-related variables included in the model development. These were categorized into four groups: demographics, preoperative, operative, and postoperative variables. MELD-Sodium score was calculated from reported preoperative sodium, bilirubin, INR, and creatinine levels according to United Network for Organ Sharing (UNOS) guidelines.^21^ Variables with a Pearson’s coefficient greater than 0.80 and/or a variance inflation factor greater than 10 were excluded due to collinearity. All variables are listed in Tables 1 and 2.

**Table 1:** Demographic and preoperative characteristics of study population

	Minor resection (n = 14,648)	Major resection (n = 6,391)	*P*
Demographics			
Age, mean (SD)	60.10 (13.62)	59.51 (13.89)	0.005
Sex			0.14
Male	7507 (51.2%)	3203 (50.1%)	
Female	7141 (48.8%)	3188 (49.9%)	
Race			0.51
White	9065 (78.3%)	3635 (77.4%)	
Black or African American	1199 (10.4%)	505 (10.8%)	
American Indian or Alaska Native	57 (0.5%)	28 (0.6%)	
Asian	1176 (10.2%)	489 (10.4%)	
Other	73 (0.6%)	38 (0.8%)	
Ethnicity, Hispanic	890 (7.4%)	356 (7.3%)	0.85
Preoperative characteristics			
BMI, mean (SD)	28.89 (6.39)	28.08 (6.08)	<0.001
Diabetes mellitus	2877 (19.6%)	1058 (16.6%)	<0.001
Smoking	2026 (13.8%)	918 (14.4%)	0.32
Functional status			0.55
Independent	14520 (99.3%)	6334 (99.4%)	
Partially dependent	93 (0.6%)	40 (0.6%)	
Totally dependent	7 (0.0%)	1 (0.0%)	
COPD	539 (3.7%)	185 (2.9%)	0.005
Ascites	49 (0.3%)	20 (0.3%)	0.90
MELD-Na, mean (SD)	8.0 (2.4)	8.2 (2.7)	<0.001
Congestive heart failure	94 (0.6%)	30 (0.5%)	0.16
Hypertension	6957 (47.5%)	2932 (45.9%)	0.032
Dialysis	49 (0.3%)	19 (0.3%)	0.76
Disseminated cancer	5790 (39.5%)	2167 (33.9%)	<0.001
Bleeding disorder	514 (3.5%)	172 (2.7%)	0.002
Preoperative transfusion	83 (0.6%)	44 (0.7%)	0.34
Preoperative septic disease			0.71
None	14514 (99.1%)	6322 (98.9%)	
SIRS	94 (0.6%)	49 (0.8%)	
Sepsis	37 (0.3%)	18 (0.3%)	
Septic shock	3 (0.0%)	2 (0.0%)	
Viral hepatitis	2024 (15.8%)	765 (13.7%)	<0.001
Tumor pathology			<0.001
Benign	3108 (21.2%)	1082 (16.9%)	
Primary hepatobiliary cancer	4398 (30.0%)	2560 (40.1%)	
Secondary (metastatic) tumor	6601 (45.1%)	2528 (39.6%)	
Neoadjuvant therapy	3923 (26.9%)	2236 (35.2%)	<0.001
Steroid use	584 (4.0%)	216 (3.4%)	0.038
Admission source			<0.001
Home	14558 (99.4%)	6346 (99.3%)	0.007
Acute or intermediate care	40 (0.3%)	21 (0.3%)	
Other	50 (0.3%)	24 (0.4%)	

*SD* standard deviation; BMI body mass index; *COPD* chronic obstructive pulmonary disease; *MELD-Na* Model for end-stage liver disease–sodium

**Table 2:** Operative and postoperative characteristics of population

	Minor resection (*n* = 14,648)	Major resection (*n* = 6,391)	*P*
Operative characteristics			
Pringle maneuver	3666 (25.0%)	1913 (29.9%)	<0.001
Concurrent intraoperative ablation	2168 (14.9%)	590 (9.3%)	<0.001
Surgical approach			<0.001
MIS	3258 (24.8%)	472 (8.1%)	
MIS with open assist	816 (6.2%)	236 (4.1%)	
Open	9037 (68.9%)	5094 (87.8%)	
Bile leakage	606 (4.2%)	679 (10.7%)	<0.001
Biliary stent	343 (2.4%)	589 (9.3%)	<0.001
Biliary reconstruction	378 (2.6%)	807 (12.8%)	<0.001
Drain(s)	5221 (35.8%)	3571 (56.1%)	<0.001
Neuraxial anesthesia	34 (0.2%)	27 (0.4%)	0.027
Operative time, mean (SD)	208.28 (100.65)	285.24 (126.10)	<0.001
Postoperative characteristics			
Need for invasive intervention Postoperatively (excluding reoperation)	811 (5.6%)	776 (12.2%)	<0.001
Posthepatectomy liver failure grade			<0.001
Grade A	103 (0.7%)	219 (3.4%)	
Grade B	62 (0.4%)	186 (2.9%)	
Grade C	16 (0.1%)	38 (0.6%)	
None	14467 (98.8%)	5948 (93.1%)	
Superficial SSI	92 (0.6%)	81 (1.3%)	<0.001
Organ Space SSI	237 (1.6%)	261 (4.1%)	<0.001
Wound dehiscence	18 (0.1%)	16 (0.3%)	0.054
Pneumonia	222 (1.5%)	148 (2.3%)	<0.001
Reintubation	74 (0.5%)	50 (0.8%)	0.020
Pulmonary embolism	59 (0.4%)	54 (0.8%)	<0.001
Failure of Ventilation Weaning	50 (0.3%)	47 (0.7%)	<0.001
Renal failure	18 (0.1%)	8 (0.1%)	1.000
UTI	105 (0.7%)	81 (1.3%)	<0.001
Stroke	8 (0.1%)	4 (0.1%)	1.000
Cardiac arrest	14 (0.1%)	16 (0.3%)	0.011
Myocardial infarction	74 (0.5%)	61 (1.0%)	<0.001
Bleeding requiring transfusion	1539 (10.5%)	1538 (24.1%)	<0.001
Deep vein thrombosis	63 (0.4%)	92 (1.4%)	<0.001
Sepsis	165 (1.1%)	149 (2.3%)	<0.001
Septic shock	33 (0.2%)	37 (0.6%)	<0.001
Reoperation	137 (0.9%)	128 (2.0%)	<0.001
Discharge destination			<0.001
Home	14209 (97.0%)	6055 (94.7%)	
Acute care	30 (0.2%)	17 (0.3%)	
Long-term facility	235 (1.6%)	155 (2.4%)	
Rehab	105 (0.7%)	109 (1.7%)	
Hospice and other	69 (0.5%)	55 (0.9%)	

*SD* standard deviation; *MIS* minimally invasive surgery; *SSI* surgical site infection; *UTI* urinary tract infection

Multivariable linear regression model was constructed first and adhered to all linear assumptions. The other AI models—RF and XGBoost—were then built to account for nonlinear relationships. Random forest models were trained with a minimum node size of 5. Hyperparameter tuning was performed by using 15-fold cross-validation to identify the optimal number of variables at each split. XGBoost models were trained with a minimum depth of each tree of 5 and learning rate (eta) of 0.05. Hyperparameter tuning was performed using 15-fold cross-validation to identify the optimal number of variables at each split. Data analyses were performed using R version 4.4.1.

### Model Performance and Inference of Nonclinical Factors

Data were randomly split into 80% training and 20% testing cohorts. Performance metrics were calculated and reported for both the training and testing data. Adjusted coefficients of determination (*R*^2^) were calculated for the MLR models to assess the impact of clinical variables on LOS within each surgical group. The *R*^2^ of the models were interpreted as the proportion of influence of clinical factors on LOS variability (e.g., an adjusted *R*^2^ value of 0.75 indicates that clinical variables account for 75% of the variability in LOS). Unlike the standard *R*^2^, the adjusted *R*^2^ accounts for the number of predictors in the model, thereby penalizing the addition of irrelevant variables and enhancing the model’s explanatory precision. For AI models, the standard *R*^2^ is reported because of the models’ complexity and nonlinear nature.^22^

Residual confounding analysis was conducted to estimate the overall importance of nonclinical factors on LOS. This approach is widely applied in epidemiological research and econometrics to evaluate the influence of unobserved confounders.^23–25^After the explanatory power of observed variables is maximized (in this case, with AI models), any remaining unexplained variability in the outcome is attributed to unobserved factors. Given the granularity of the ACS-NSQIP hepatectomy-targeted dataset and the inclusion of variables that have predicted with extremely high accuracy postoperative outcomes, the unmeasured effects were safely presumed to be primarily nonclinical.

Mean absolute error (MAE) was also measured, providing an indication of the average prediction error in days between patients’ predicted and actual LOS (e.g., MAE of two means that, on average, the model’s predictions deviate by about two days from the actual observed values).

### Secondary Analyses

To gain insight into the model and to determine the contribution of each clinical variable in explaining LOS, Increase in Node Purity metric was used for the highest performing AI model. A sensitivity analysis was performed to assess the variability in LOS in a “best-case scenario” among patients with no postoperative complications.

### Missing Data

Variables with more than 10% missing values were imputed using Multiple imputation by Chained Equations (MICE). All models were trained on five imputed datasets, and the results were pooled to produce the final analysis. Missing data for each variable are presented in Supplementary Table 1.

### Data Sharing

ACS-NSQIP is a de-identified database accessible via ACS website at https://facs.org upon request. The data is available to all participating hospitals in the ACS-NSQIP program. Users must adhere to the data use agreement, provide contact details, and complete a brief online questionnaire. The data dictionary, detailing the variables in the dataset, is available on the ACS website.

## Results

### Population Demographics

**Fig. 1:**
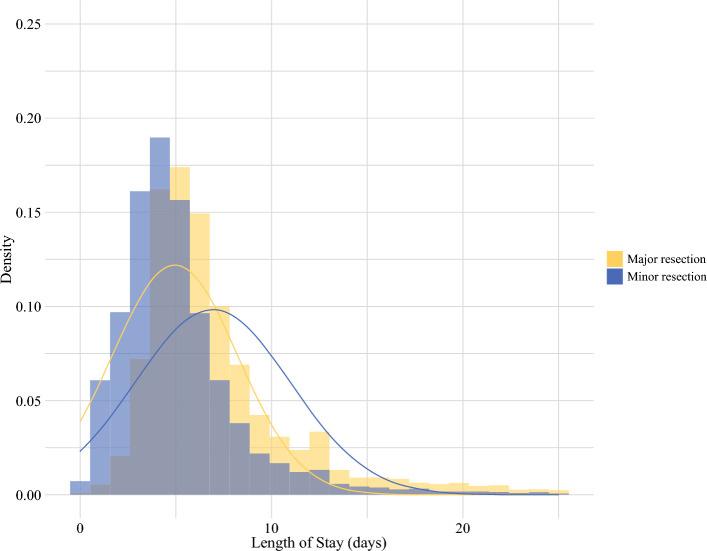
Length of stay distribution by surgical procedure type. Patients undergoing minor resection had longer and more variable postoperative length of stay than major resection

A total of 21,039 patients were included (mean age: 60 years; 51% male): 14,648 (70%) underwent minor resection, and 6,391 (30%) underwent major resection. The clinical characteristics are shown in Tables 1 and 2: the mean age of the cohort was 60 years; 51% were male; 67% of resections were open approach; and the mean MELD-Na score was 8.0 (standard deviation [SD] 2.5). The mean LOS was longer for major resection (6.9 days [SD 4.1]) than minor resection (5.0 days [SD 3.3]) (Fig. 1).

### Model Comparison

**Fig. 2:**
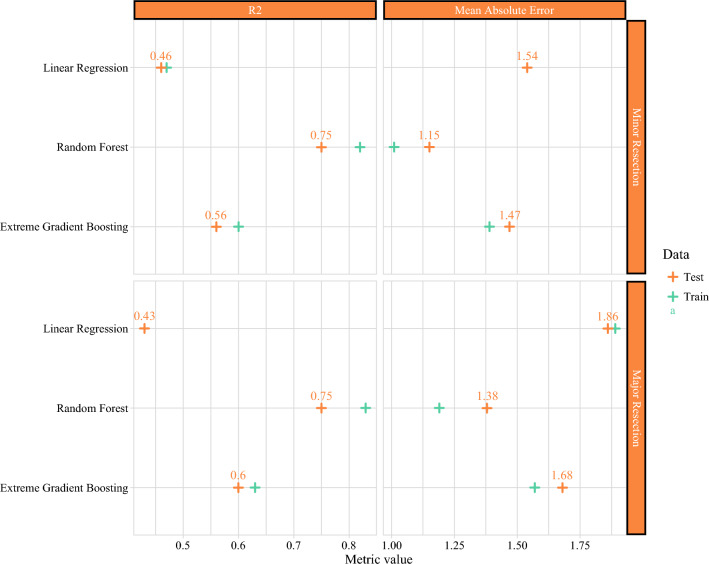
Performance metrics for predicting length of stay by surgical procedure type. Multivariable linear regression yielded R-squared (*R*^2^) values of 0.46 for minor resection and 0.43 for major resection on testing data. AI models accounting for nonlinear relationships markedly enhance *R*^2^ for both minor and major resections; increase from 0.46 to 0.75 for minor resection and from 0.43 to 0.75 for major resection. The mean absolute error indicated an average unexplained length of stay of 1.15 to 1.54 days for minor resection and 1.38 to 1.86 days for major resection. Standard *R*^2^ values are reported for random forest and extreme gradient boosting due to model complexity

**Fig. 3:**
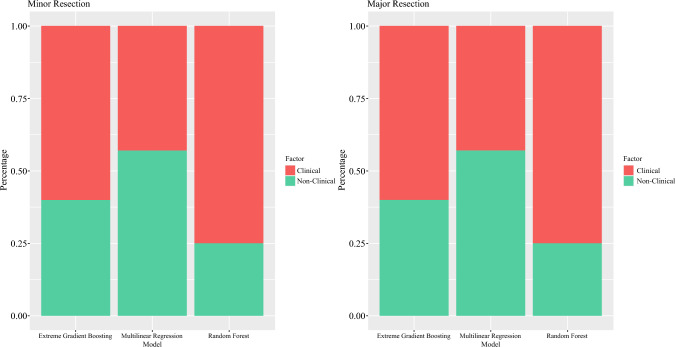
Clinical vs. nonclinical factors influence on LOS. Factors determined by *R*^2^ from each model by surgical group. Random Forest maximized the clinical factor influence on LOS with an *R*^2^ of 75% for both surgical cohorts, inferring a nonclinical influence of 25% by residual confounding analysis

Figure 2 shows the performance metrics of each model per surgical group. The adjusted *R*^2^ for the MLR models were 0.46 for minor resection and 0.43 for major resection, suggesting that 46% and 43% of patients’ LOS after minor and major resection is attributed to the linear and additive effects of clinical factors. The performance of AI models was significantly higher compared with MLR, indicating the importance of nonlinear variable interaction in explaining LOS. The highest performing model was RF with *R*^2^ of 0.75 for both groups, outperforming MLR and XGBoost models. This indicates that clinical factors, when accounting for complex nonlinear interactions, explain 75% of LOS variability after hepatectomy, thus representing the true extent of clinical factors’ influence on LOS. Given the nonparsimonious approach to model building and the maximizing of the explanatory power of clinical and patient-related variables through hyperparameter tuning, we infer that the residual unexplained variability (i.e., 25%) is due to the influence of unmeasured nonclinical factors (Fig. 3).

We then calculated the mean absolute error (MAE), which represents the difference between actual and model-predicted LOS to determine the number of days that could not be explained by clinical factors alone and that are therefore due to the influence of nonclinical variables. Results from the MAE analysis showed that clinical factors could not explain 1.15 to 1.54 days of patients’ LOS after minor resection, as well as 1.38 to 1.86 days after major resection. Residual plots showed the difference between model-predicted, and patients’ actual LOS followed a normal distribution centered near zero, indicating that our models were unbiased, neither systematically overestimating nor underestimating LOS (Supplementary Fig. 1).

### Impact of Variables on LOS

**Fig. 4:**
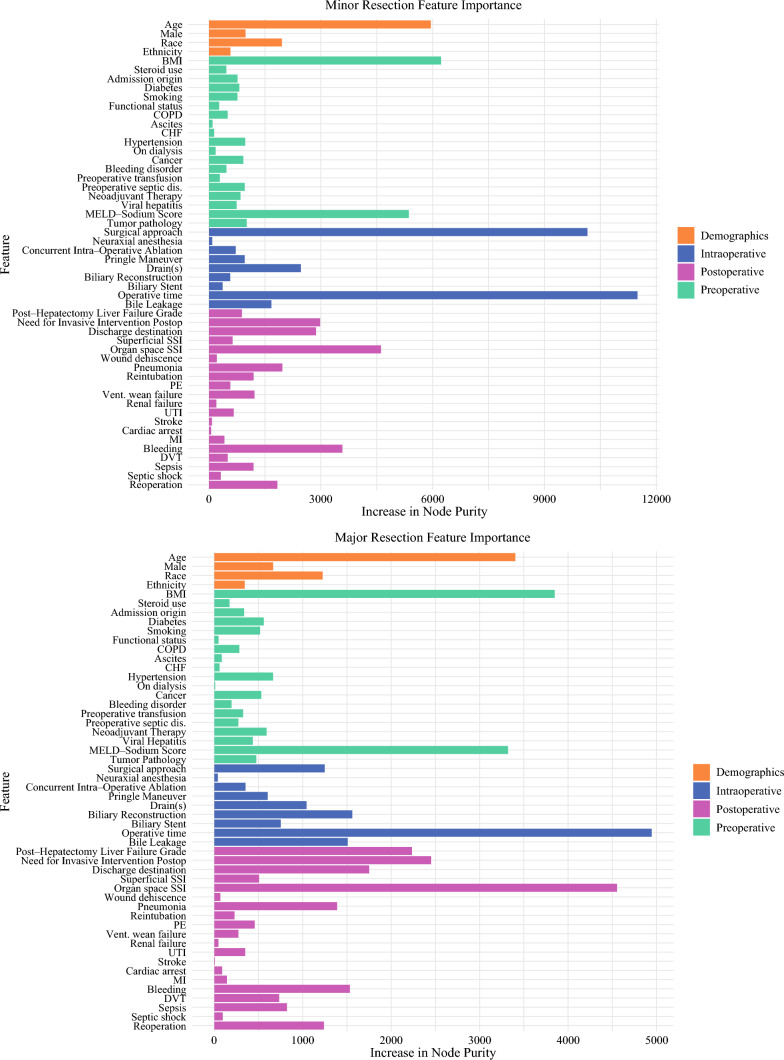
Variable importance for predicting length of stay using random forest models across surgery groups. For minor resection, intraoperative factors had the greatest overall importance on LOS variability, then postoperative, preoperative, and demographic factors in decreasing importance. Individually, the top five influential variables were operative time, surgical approach, BMI, age, and MELD-Na score. The notable complications in minor resection were organ space SSI and bleeding. For major resection, postoperative factors had the greatest overall importance, then preoperative, intraoperative, and demographic factors in decreasing importance. Individually, the top five influential variables were operative time, organ SSI, BMI, age, and MELD-Na Score. The high importance of MELD-Na score aligns with existing research^26^

Feature importance analysis was conducted to identify which clinical and patient-related variables contributed most significantly to explaining patients’ LOS. Overall, intraoperative and postoperative factors were more influential than demographic and preoperative characteristics (Fig. 4). Specifically, intraoperative time had the highest impact on LOS variability among clinical factors in patients undergoing major and minor resection. Other influential factors include age, BMI, MELD-Na score, organ space infection (for major resection), and surgical approach (for minor resection).

**Fig. 5:**
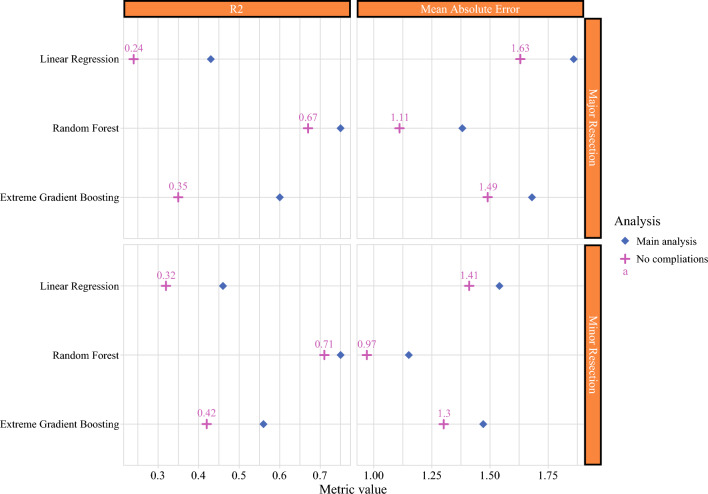
Performance metrics for predicting length of stay by surgical procedure type for sub-analysis of patients with no complications. Compared with the main analysis, *R*^2^ and MAE decreased in patients with no complications when compared to the main analysis for all models. Note. The *R*^2^ for random forest decreased marginally by 8% and 4%, for minor and major resection, respectively

Given that postoperative outcome had a substantial impact on LOS among clinical variables especially for major resection, a sensitivity analysis was conducted analyzing patients with ideal postoperative course, who did not develop any complication. This analysis revealed a lower MAE (0.97–1.41 days for minor resection; 1.11–1.63 days for major resection), and lower *R*^2^ for both surgical groups (0.71 for minor resection; 0.67 for major resection) compared with the primary analysis. This suggests that while the absence of complications is associated with a shorter LOS and reduced MAE, the role of clinical factors in explaining LOS variability diminishes, indicating that nonclinical factors have a greater influence on LOS variability in these patients (Fig. 5).

## Discussion

While the impact of nonclinical factors on LOS is acknowledged, their precise influence relative to that of clinical variables remains largely unquantified. This is due to two main challenges: the difficulty in measuring all nonclinical factors, and the limitations of traditional linear models to capture the true influence of clinical factors on LOS as they cannot account for complex variable interactions. This gap can lead to inefficient resource allocation as it may result in efforts yielding smaller returns by incorrectly targeting factors that have smaller than anticipated influence on LOS. This study addresses this gap by developing a novel AI framework to accurately quantify the proportion of LOS variability attributable to clinical factors in major and minor liver resections and estimate the overall influence of nonclinical factors from the residuals. This approach also enables us to consider the distinction between actionable and nonactionable factors, which could further assist hospitals in identifying areas where intervention might be feasible. While many clinical factors may be less modifiable, certain nonclinical factors (e.g., discharge planning processes, access to postacute resources) may be more adjustable and offer a pathway to reducing LOS. One way to conceptualize the distinction is by viewing nonclinical factors as requiring “higher activation energy”—they may require more initial effort to address, but once targeted, their impact is broader and more sustainable across the patient population. In contrast, clinical factors have a “lower activation energy,” because they often require specific changes per patient but may not produce as wide-reaching benefits due to variability in patient compliance and other individual factors. By understanding this distinction, hospitals can prioritize improvements that leverage the more modifiable, system-level nonclinical interventions to yield significant and lasting reductions in LOS. In addition to informing where efforts should be focused to improve surgical quality of care, this study provides a valuable reference for physicians and hospitals interpreting benchmarking results. These benchmarks are typically risk-adjusted only for clinical factors, omitting the influence of nonclinical factors, such as transportation, logistical delays, and patient preferences on LOS. Understanding the balance of the influence of clinical and nonclinical factors provides a context with which to interpret the results.

We leveraged the ACS-NSQIP database since it is a recognized tool for benchmarking hospital performance against national standards and for assessing the effectiveness of quality improvement initiatives.^27,28^ Additionally, the database has remarkably complete data collection and provides a large sample size, increasing the power and generalizability of this study’s findings.^29^ NSQIP’s hepatectomy-targeted participant use file (PUF) includes a comprehensive set of variables that are most clinically relevant to outcomes following liver surgery.^30^

Our study found that 75% of the variability in LOS after hepatectomy is explained by clinical and patient-related factors. This finding aligns with the nature of liver resection as a complex, high-risk procedure typically performed in specialized tertiary care centers, which have implemented standardized postoperative recovery protocols and streamlined admission-to-discharge pipelines.^2^ Furthermore, the elective nature of hepatectomies facilitates careful preoperative preparation, allowing for early initiation of discharge planning. This contrasts with other surgical fields. For instance, colectomies, performed by both general and colorectal surgeons, may see more significant variability in postoperative management given the sometimes more emergent nature of the operations.^31^ Similarly, in Trauma and Emergency General Surgery, where the unpredictable nature of cases makes it challenging to anticipate discharge timing, nonclinical factors significantly impact LOS.^18,20^

While our findings highlight the significant role of clinical factors in determining LOS after hepatectomy, it is crucial to interpret these results in the proper context. The higher proportion of LOS explained by clinical variables should not be misconstrued to mean that patients who undergo hepatectomy have less complicated or better postoperative courses than those undergoing other procedures. On the contrary, major complications are common and can be devastating after hepatectomy.^32–37^ In our study, a substantial percentage of patients developed complications after surgery, including serious outcomes such as bile leakage, infections, and respiratory distress. The specific risk of postoperative liver failure, one of the most serious complications, ranges from 0.7% to 9.1%, depending on the extent of liver resection and the patient’s remaining liver function.^38^ However, even with these complications, the proportion of clinical variability in LOS remains higher than in other fields.^18^ This suggests that once complications are resolved, there is minimal logistical delay in discharging patients from the hospital, unlike in other surgical specialties. Thus, while the proportion of clinical variability explaining LOS is influenced by complications and patients’ preoperative status, logistical and administrative delays, such as bed availability, staffing issues, and surgeon preference, play a less prominent role compared with other fields. This efficient management of nonclinical factors further underscores the benefits of the specialized care and standardized processes in hepatobiliary surgery centers discussed earlier.

Among clinical factors, organ space surgical site infection (SSI), bleeding, liver failure, and need for invasive intervention emerged as the primary drivers of LOS. Previous studies in other fields show that complications delay the discharge process, potentially increasing the influence of nonclinical drivers.^39,40^ To investigate this phenomenon in hepatectomy, we examined LOS in a “best-case scenario” subset of patients who experienced no postoperative complications. In this cohort, the mean absolute error (MAE) and *R*^2^ decreased for both surgical groups across all models. Notably, while the *R*^2^ decreased in random forest models, the reduction was marginal, still maintaining a high *R*^2^ at 67% and 71% for minor and major resections, respectively. This suggests that the absence of complications is associated with a shorter LOS and a reduced MAE. As such, the proportion of LOS variability explained by clinical factors is still predominate over nonclinical factors, but to a lesser extent.

The strength of our approach lies in the application of advanced AI techniques. Previous studies on LOS predictors primarily rely on linear models, neglecting nonlinear interactions of variables. However, this assumption does not reflect clinical reality, where clinical factors may gain or lose significance depending on the presence or absence of other variables. For example, the impact of advanced age, MELD-Na score, and postoperative complications like bile leakage on LOS may be significantly different when considered together rather than in isolation. To better capture these interactions, we developed two different AI models—random forest and extreme gradient boosting—which were fine-tuned to maximize the explanatory power of clinical factors on LOS. Interestingly, we found that the performance metrics of our advanced AI models outperform those of traditional linear regression, suggesting that the remaining discrepancy between predicted and actual LOS is due to nonlinear relationships rather than nonclinical factors.

This study has several limitations that warrant consideration. First, while the ACS-NSQIP hepatectomy-targeted database is comprehensive, it may not capture all possible clinical factors affecting LOS. However, we believe the influence of any missing clinical factors is likely minimal, given that our analysis includes many well-established predictors of postoperative outcomes, demonstrating high accuracy.^41–46^ The unexplained variability in our model is therefore more likely to stem from unmeasured nonclinical factors rather than omitted clinical variables. This conclusion is supported by the use of a hepatectomy-specific database, the inclusion of robust clinical predictors, and the application of machine learning techniques capable of capturing complex, nonlinear relationships among these variables. One notable limitation of the current dataset is the absence of direct measurements of hospital-level variables, such as resource availability, operational protocols, and institutional policies, which may contribute to the observed variability in LOS. These variables are rarely, if ever, captured in large registries. However, including hospital identifiers could enable differentiation between hospital-level factors and other nonclinical, nonhospital factors. Unfortunately, this variable is not available in NSQIP, which prevents us from conducting such an analysis. Future studies incorporating hospital identifiers are essential to better distinguish hospital-level influences from other nonclinical contributors to LOS variability. Additionally, nonclinical factors, such as insurance status and other social determinants, were not included due to dataset constraints. These factors may contribute to the unexplained variability in LOS, as socioeconomic factors often influence access to postoperative resources and continuity of care, both of which can significantly impact discharge planning.

Second, hepatectomies are less common compared to other surgical procedures, resulting in a smaller sample size. This introduces potential limitations on the generalizability and precision of the study. However, we chose RF and XGBoost as they are incredibly versatile and reliably perform with good accuracy even with small training datasets.^47,48^Additionally, we tailored the number of predictors to adhere with the conservative “factor 50” rule-of-thumb recommended for AI models.^49^ Finally, our study faced challenges related to missing data. While the proportion of missing data for each individual variable did not exceed 26.5%, the number of patients with no missing variables across all fields was exceptionally small. This limitation prohibited us from conducting a complete case analysis to assess the potential impact of multiple imputation on our results.

Future studies should focus on directly measuring nonclinical factors and comparing them with the insights from our models. While research in other fields has examined the isolated impact of nonclinical factors on LOS—such as bed availability, for-profit hospital status, psychosocial factors, and hospital staffing—these studies only begin to reveal their combined effects on patient LOS.^50–54^ Moreover, research specifically addressing LOS after hepatectomy is limited, highlighting the need for further investigation. Expanding data collection to include system-related and structural variables could offer a more accurate understanding of the factors influencing LOS after hepatectomy, validate our findings, and ultimately lead to more effective strategies for improving surgical outcomes.

This study demonstrates the effectiveness of an AI-driven framework in quantifying the impact of clinical factors on LOS after hepatectomy, attributing 75% of LOS variability to these factors. The remaining 25% of variability, inferred to be due to nonclinical factors, underscores the importance of capturing system-related variables for a more complete understanding of LOS determinants. These findings highlight the multifactorial nature of posthepatectomy recovery and the need for comprehensive data collection to improve quality benchmarking in surgical care. Future research should aim to directly measure nonclinical factors to validate these inferences and enhance surgical outcome strategies.

## Supplementary Information

Below is the link to the electronic supplementary material.

Supplementary file1 (PDF 329 kb)
