# Supplementary material for: Developing a Novel Artificial Intelligence Framework to Measure the Balance of Clinical Versus Nonclinical Influences on Posthepatectomy Length of Stay
Source: Ann Surg Oncol. 2025 Feb 5;32(5):3526–38. doi: 10.1245/s10434-025-16942-5 (PMC11976349; doi:10.1245/s10434-025-16942-5)
Supplement: Supplementary file 1 — Supplementary file1 (PDF 329 kb) [file 10434_2025_16942_MOESM1_ESM.pdf]

SUPPLEMENTARY FIGURES

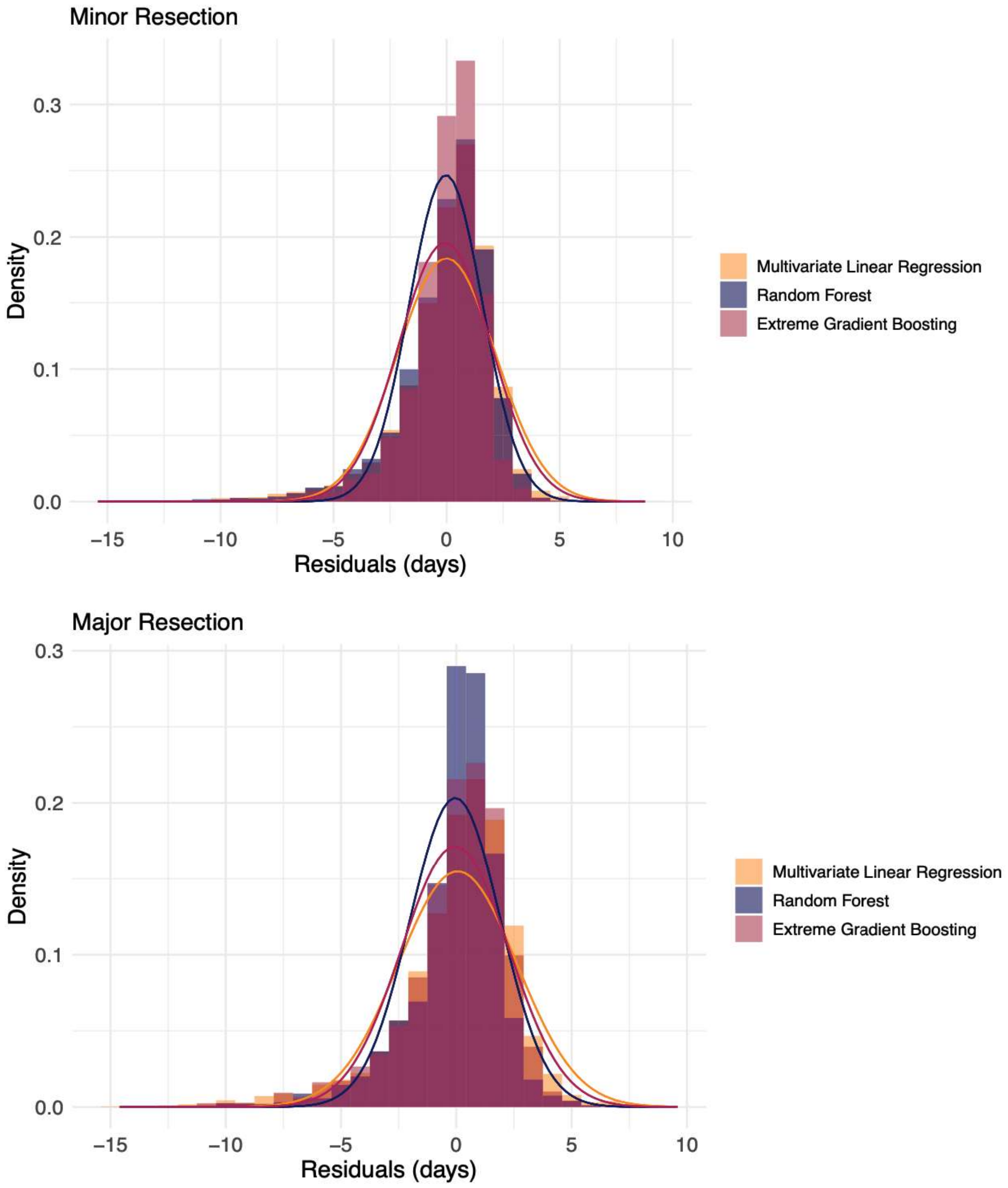

Supplementary Figure 1. Length of stay residual plot for each model. Residual plots showed the difference between model-predicted, and patients 'actual LOS followed a normal distribution centered near zero.

Table 1. Data missingness amongst variables.

	Minor Resection (n = 14,648)	Major Resection (n=6,391)
**DEMOGRAPHICS**		
Age	0 (0%)	0 (0%)
Sex	0 (0%)	0 (0%)
Race	3078 (21.0%)	1696 (26.5%)
Ethnicity, Hispanic	2610 (17.8%)	1511 (23.6%)
**PREOPERATIVE CHARACTERISTICS**		
BMI	99 (17.8%)	40 (0.62%)
Diabetes Mellitus	0 (0%)	0 (0%)
Smoking	0 (0%)	0 (0%)
Functional Status	28 (0.2%)	16 (0.3%)
COPD	0 (0%)	0 (0%)
Ascites	0 (0%)	20 (0.3%)
MELD-Na*		
Sodium	647 (4.4%)	224 (3.5%)
Creatinine	541 (3.7%)	182 (2.9%)
Bilirubin	1122 (7.7%)	413 (6.5%)
INR	2408 (16.4%)	729 (11.4%)
Congestive Heart Failure	0 (0%)	0 (0%)
Hypertension	0 (0%)	0 (0%)
Dialysis	0 (0%)	0 (0%)
Disseminated Cancer	0 (0%)	0 (0%)
Bleeding Disorder	0 (0%)	0 (0%)
Preoperative Transfusion	0 (0%)	0 (0%)
Preoperative Septic Disease	0 (0%)	0 (0%)
Viral Hepatitis	1817 (12.4%)	791 (12.4%)
Tumor Pathology	541 (3.7%)	221 (3.5%)
Neoadjuvant Therapy	85 (0.6%)	30 (0.5%)

Steroid Use	0 (0%)	0 (0%)
Admission Source	0 (0%)	0 (0%)
**OPERATIVE CHARACTERISTICS**		
Pringle Maneuver	0 (0%)	0 (0%)
Concurrent Intra-Operative Ablation	81 (0.6%)	32 (0.5%)
Surgical Approach	1537 (10.5%)	589 (9.2%)
Bile Leakage	113 (0.8%)	51 (0.3%)
Biliary Stent	104 (0.7%)	42 (0.7%)
Biliary Reconstruction	113 (0.8%)	76 (1.2%)
Drain(s)	52 (0.4%)	28 (0.4%)
Neuraxial Anesthesia	31 (0.2%)	3 (0.05%)
Operative Time	2 (0.01%)	1 (0.02%)
**POSTOPERATIVE CHARACTERISTICS**		
Need for Invasive Intervention Postoperatively (excluding reoperation)	54 (0.4%)	20 (0.3%)
Post-Hepatectomy Liver Failure Grade	0 (0%)	0 (0%)
Superficial SSI	0 (0%)	0 (0%)
Organ Space SSI	0 (0%)	0 (0%)
Wound Dehiscence	0 (0%)	0 (0%)
Pneumonia	0 (0%)	0 (0%)
Reintubation	0 (0%)	0 (0%)
Pulmonary Embolism	0 (0%)	0 (0%)
Failure of Ventilation Weaning	0 (0%)	0 (0%)
Renal Failure	0 (0%)	0 (0%)
UTI	0 (0%)	0 (0%)
Stroke	0 (0%)	0 (0%)
Cardiac Arrest	0 (0%)	0 (0%)
Myocardial Infarction	0 (0%)	0 (0%)
Bleeding Requiring Transfusion	0 (0%)	0 (0%)
Deep Vein Thrombosis	0 (0%)	0 (0%)
Sepsis	0 (0%)	0 (0%)

Septic Shock	0 (0%)	0 (0%)
Reoperation	0 (0%)	0 (0%)
Discharge Destination	0 (0%)	0 (0%)

*Report missingness of preoperative sodium, INR, bilirubin, and creatinine lab result as required for MELD-Na calculation
